# Supplementary figures and images for: Fluorescence fluctuation analysis reveals PpV dependent Cdc25 protein dynamics in living embryos
Source: PLoS Genet. 2020 Apr 6;16(4):e1008735. doi: 10.1371/journal.pgen.1008735 (PMC7162543; doi:10.1371/journal.pgen.1008735)

Supplementary data Figure S1

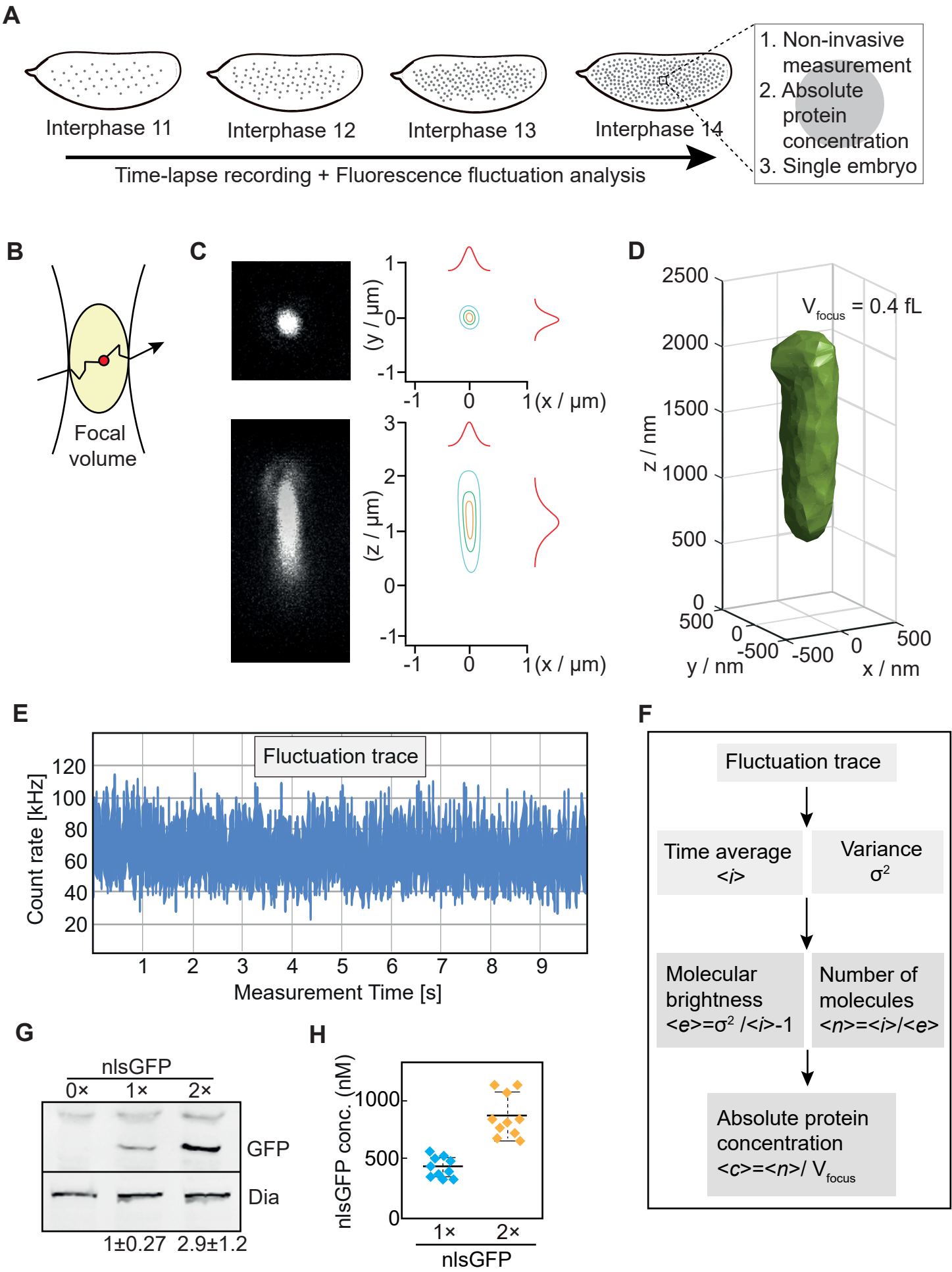

Supplement: S1 Fig — (A) Scheme of fluorescence fluctuation analysis application in living embryo. (B) Principle of fluorescence fluctuation analysis. Passing through the focal volume leads to changes in the fluorescence signal. The frequency of these changes depends on the concentration. (C) Isosurface representations of a microsphere. (D) The volume of the point spread function. (E) Fluctuation trace of a measurement (10 seconds). (F) Time average and variance are computed from the fluctuations trace, which allows calculation of average molecular brightness and average number of molecules within the focal volume, as well as absolute protein concentration. (G) Western blot with extracts of the embryos (0–3 h) with 0×, 1×, 2× copies of nlsGFP transgene. Loading control by Dia protein, which is a ubiquitously expressed formin. Quantification by densitometry (N = 3, biological replicates on the same blot, average with standard deviation). (H) Concentration per focal volume as determined by fluorescence fluctuation analysis of GFP fluorescence. Mean, bold line. Standard deviation, dashed line. Source data are listed in S1 Data. (PDF) [file pgen.1008735.s001.pdf]
