# Supplementary material for: Fluorescence fluctuation analysis reveals PpV dependent Cdc25 protein dynamics in living embryos
Source: PLoS Genet. 2020 Apr 6;16(4):e1008735. doi: 10.1371/journal.pgen.1008735 (PMC7162543; doi:10.1371/journal.pgen.1008735)

Supplementary data Figure S2. Exponential fitting of fluctuation analysis measurements

Twine-GFP/+

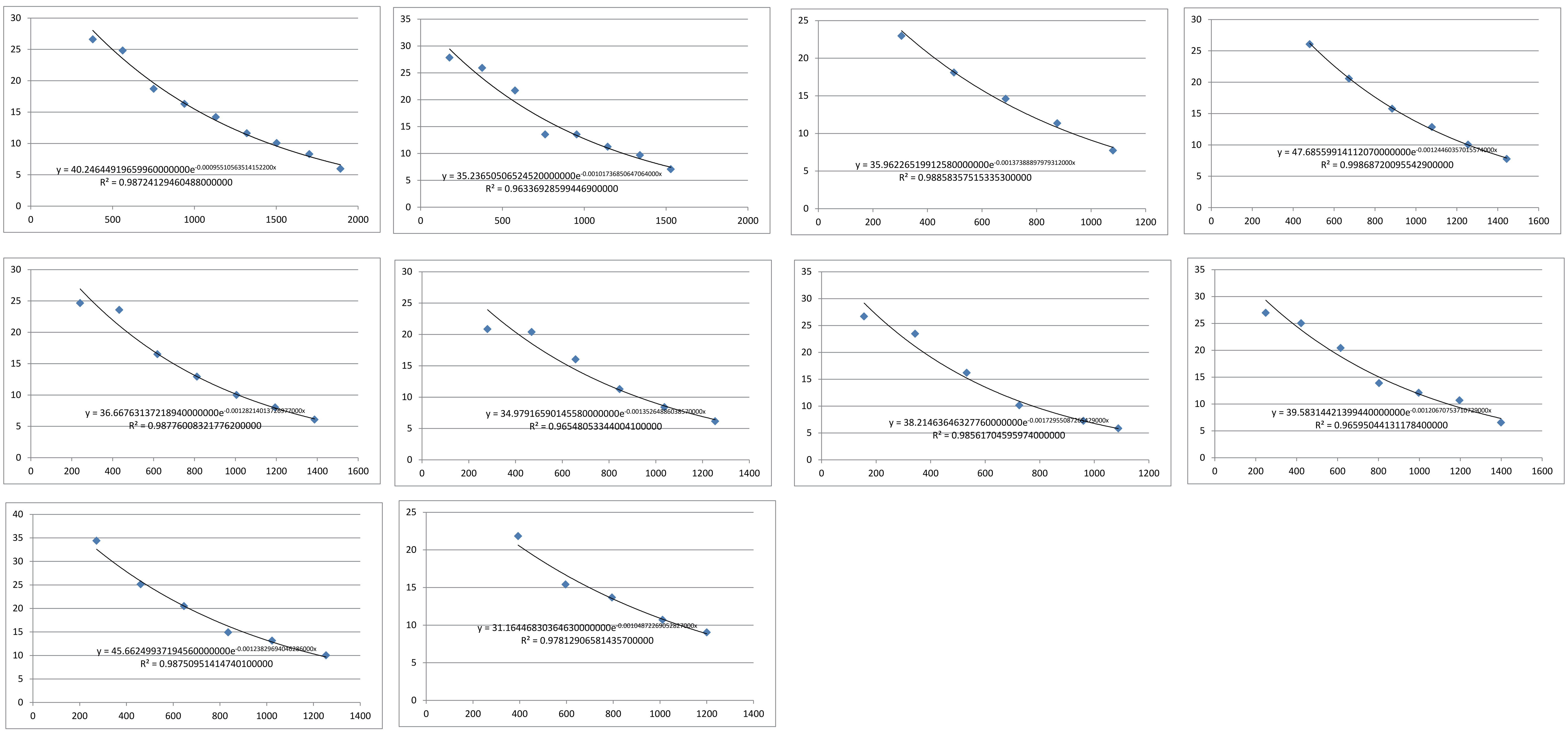

PpV; Twine-GFP/+

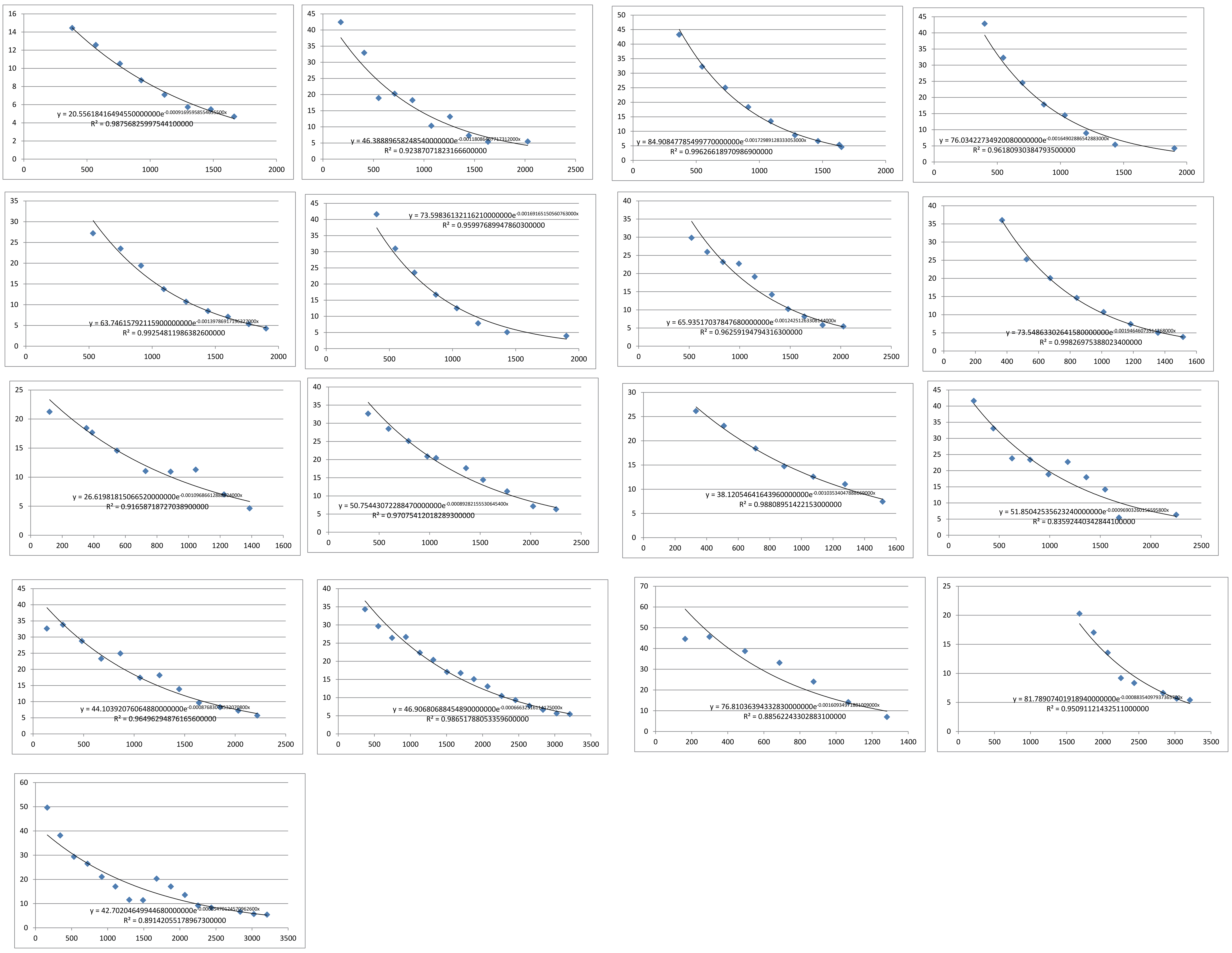

tribbles, Twine-GFP/tribbles

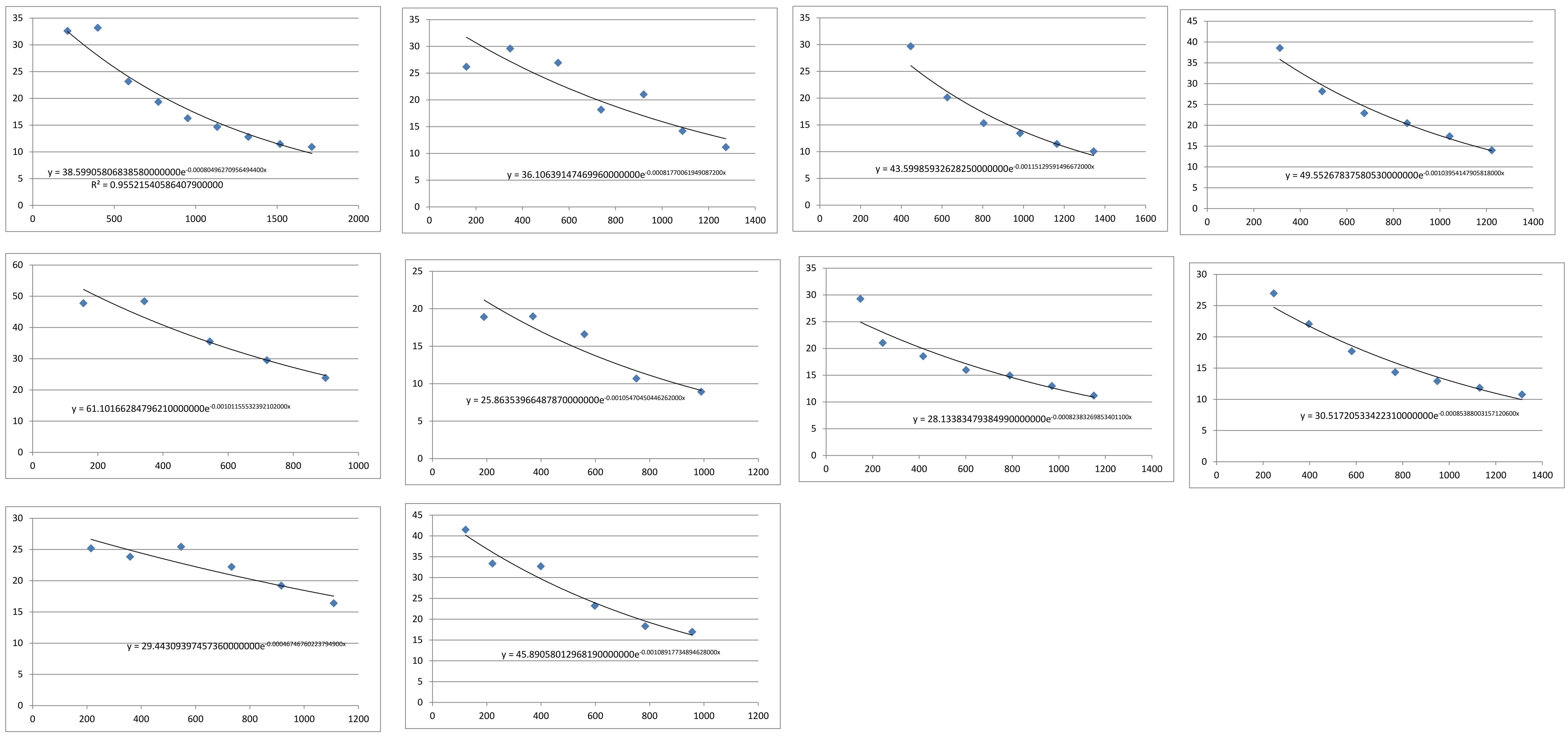

Supplement: S2 Fig — Exponential fitting of fluorescence fluctuation analysis measurements. Formulas representing fitting curve of exponential trend line c(t) = c0 · e-kt, and coefficient of determination value R2. Unit of the x-axis is second. (PDF) [file pgen.1008735.s002.pdf]
