# Supplementary material for: Fluorescence fluctuation analysis reveals PpV dependent Cdc25 protein dynamics in living embryos
Source: PLoS Genet. 2020 Apr 6;16(4):e1008735. doi: 10.1371/journal.pgen.1008735 (PMC7162543; doi:10.1371/journal.pgen.1008735)
Supplement: S1 Table — The average peak area of five peptides were determined by XIC (extracted ion chromatograms) and compared between the wild type (wt) and the PpV mutant samples. The AA ratio of wt/PpV averages at 1.78 indicating that non-phosphopeptides were consistently about 1.8-fold more abundant in the wild type compared to PpV mutant samples. (DOCX) [file pgen.1008735.s003.docx]

| **Peptide** | **mass** | **AA (wt)** | **AA (*PpV*)** | **AA ratio (wt/*PpV*)** |
| --- | --- | --- | --- | --- |
| ALGDEPELIGDLSK | 728.8799 | 18358242 | 8486655 | 2.16 |
| LIQGEFDEQLGSQGGYEIIDCR | 843.0633 | 2797579 | 1879327 | 1.49 |
| YPYEFLGGHIR | 676.3420 | 40374669 | 22168420 | 1.82 |
| IYVFHCEFSSER | 787.3584 | 8807155 | 5181341 | 1.7 |
| GQIQEAFPTLTSNQENR | 966.9741 | 44055348 | 25160476 | 1.75 |

**S1 Table**
