# Supplementary material for: Fluorescence fluctuation analysis reveals PpV dependent Cdc25 protein dynamics in living embryos
Source: PLoS Genet. 2020 Apr 6;16(4):e1008735. doi: 10.1371/journal.pgen.1008735 (PMC7162543; doi:10.1371/journal.pgen.1008735)
Supplement: S2 Table — Results from Mascot data analysis of mass spectrometry (ms/ms) spectra. Peptide sequences are depicted of the Cdc25/Twine as predict from fragmentation spectra. Predicted phosphorylation sites are marked in red (bold colors for unambiguous annotations). Although peptides were identified independently in many cases only highest scoring peptides are included. Observed mass/charge (M/Z) values indicate the result of the measurement and the calculated relative molecular weight (Mr) from the M/Z is indicated as experimental (expt) Mr in Dalton (Da). Mr calc depicts the calculated relative molecular weight in Dalton (Da) as calculated from the expected Mr from the database. Ppm indicates the error value between Mr expt and Mr calc and the ion score indicates the number of spectral ions matching the annotated fragments in the database. The expectation value is a statistical representation of the ion score expressed as p value (Student’s t-test). (DOCX) [file pgen.1008735.s004.docx]

**S2 Table**

| Wild type |  |  |  |  |  |  |  |
| --- | --- | --- | --- | --- | --- | --- | --- |
| **Peptides** | **Position** | **M/Z** | **Mr (expt)** | **Mr(calc)** | **Ppm** | **Ion score** | **Expect** |
| K.**S**WQCGEGGDSGIGGGGSR.G | S396 | 902.3405 | 1802.6665 | 1802.668 | -0.83 | 46 | 4.40E-05 |
| K.TKSWQCGEGGDSGIGGGGSR.G | T394, S396 | 1016.9122 | 2031.8099 | 2031.8106 | -0.34 | 68 | 4.30E-07 |
| K.TL**S**MNDAEIMR.A | S205 | 696.7799 | 1391.5453 | 1391.5462 | -0.66 | 32 | 0.00094 |
| K.TLSMNDAEIMR.A | T203, S205 | 696.7805 | 1391.5464 | 1391.5462 | 0.13 | 31 | 0.004 |

| *PpV* mutant |  |  |  |  |  |  |  |
| --- | --- | --- | --- | --- | --- | --- | --- |
| **Peptides** | **Position** | **M/Z** | **Mr expt (Da)** | **Mr calc (Da)** | **Ppm** | **Ion score** | **Expect** |
| K.SWQCGEGGDSGIGGGG**S**R.G | S412 | 902.3414 | 1802.6682 | 1802.668 | 0.12 | 47 | 8.50E-05 |
| R.KTL**S**MNDAEIMR.A | S205 | 744.833 | 1487.6515 | 1487.6513 | 0.11 | 62 | 3.70E-06 |
| K.SWQCGEGGD**S**GIGGGGSR.G | S405 | 902.3408 | 1802.6671 | 1802.668 | -0.49 | 41 | 0.00014 |
| R.RK**S**AVQETPLQWMLK.R | S41 | 632.3252 | 1893.9538 | 1893.9536 | 0.11 | 27 | 0.0032 |
| R.KTLSMNDAEIMR.A | T203, S205 | 752.8309 | 1503.6473 | 1503.6462 | 0.72 | 33 | 0.00078 |
| K.TKSWQCGEGGDSGIGGGGSR.G | T394, S396 | 1016.9127 | 2031.8109 | 2031.8106 | 0.14 | 29 | 0.0073 |
| HIPASTTVLSPITELSQNMNGAR | S59, T60, T61, S64, T67 |  | 2532.2043 | 2532.2043 | 0.42 |  |  |
