# Supplementary material for: Fluorescence fluctuation analysis reveals PpV dependent Cdc25 protein dynamics in living embryos
Source: PLoS Genet. 2020 Apr 6;16(4):e1008735. doi: 10.1371/journal.pgen.1008735 (PMC7162543; doi:10.1371/journal.pgen.1008735)
Supplement: Attachment — Submitted filename: Review_twe dynamics.pdf [file pgen.1008735.s007.pdf]

Comments on 'FFA reveals Ppv dependent Cdc25 protein dynamics in living embryos'

Originality: To my knowledge the application of FFA to developmentally assess Twe levels and degradation kinetics during cycle 14 is novel and represents an excellent case study for this technology. Previous work indicated that additional factors besides Trbl contribute to Twe destabilization. Evidence that Ppv represents such an additional factor is examined in this study.

Importance to field: Unclear. How Cdc25 (both Twe and Stg) phosphatases affect Cdk1 regulation of mitosis during syncytial cycles and the maternal-zygotic transition has been intensively studied in Drosophila. The authors propose that Ppv-mediated Twe turnover during late syncytial cycles sets a threshold level that contributes to rapid degradation of Twe during early cycle 14, independently of Trb (or other) mechanisms. Data showing differences in Twe concentration between control and *Ppv* genotypes and identification of Twe phospho-peptides unique to *Ppv* mutants is consistent with their model. I don't see how the experiments with transgenic mutant forms of Twe support this as an essential mechanism, however. Changes in phosphorylation status apparently did not compromise either mutant transgene's ability to complement *twe* mutant female sterility and both mutants weakly promoted extra syncytial cycles, as a measure of Twe activity, but it wasn't that much of an effect.

Broad interest to genetics and genomics: Potentially this method for dynamically quantitating protein levels in living embryos will be useful for many researchers. The issues they are dealing with are broadly applicable and relevant to understanding other inherently variable processes.

Rigorous methodology: Not being familiar with the FFA technique, I cannot comment on that aspect. It wasn't clear to me how the rate curves were generated, but I appreciated seeing the spread of the individual embryo data. By using a Twe-GFP transgene for these studies in a context of two normal *twe* alleles the overall Cdc25 levels were effectively increased. Is there a way to account for effects related to Cdc25 activity levels?

Substantial evidence for conclusions: While the data were convincing, in my view the results were too broadly interpreted to uphold the conclusions stated in the Abstract.

Minor points:
It seems misleading to say (line 58) that nuclear cycles proceed with 'ultrafast S phase of about 5 minutes' when they range from ~2 to 20 minutes long.

Needs editorial improvement in places, for example: 36_the pause is occurred

I don't think you can accurately say (69-70) that 'only Twe is required for nuclear cycles', given the conclusions of references [18, 19].

Wee1 phosphorylates Cdk1 on Y15. The Wee1-related Myt1 kinase phosphorylates Cdk1 on T14 and Y15.

Fig 1B/C: the evidence that Twe disappears more slowly in *Ppv* mutants vs WT in cycle 14 by IF looks convincing (for the two embryos shown) but the westerns showed little difference between these genotypes. Should the western data be discounted because of inherent difficulty with normalizing embryo lysates? High embryonic variability seems to be a feature of *Ppv* mutant embryos, making it difficult to draw conclusions from these comparisons.

Fig 4. The proteomics analysis of Twe-GFP shows a big difference by NC11 in Twe concentration between the different transgenes. Phospho-mimetic mutant has much lower levels at all time points – does this mean the changes are destabilizing the protein but also making it refractory to further loss below this threshold? Phospho-acceptor mutant starts higher, but seems to decline at a similar (?) rate as WT. Yet both transgenes rescued *twe* mutant female sterility. Do these results really support Ppv-mediated turnover of Twe being important for MZT kinetics?
