# Supplementary material for: Fluorescence fluctuation analysis reveals PpV dependent Cdc25 protein dynamics in living embryos
Source: PLoS Genet. 2020 Apr 6;16(4):e1008735. doi: 10.1371/journal.pgen.1008735 (PMC7162543; doi:10.1371/journal.pgen.1008735)
Supplement: Attachment — Submitted filename: Review PlOS Genetics 12-2019.docx [file pgen.1008735.s006.docx]

Fluorescence fluctuation analysis (FFA) reveals PpV dependent Cdc25 protein dynamics in living embryos.

In this paper, the authors have analyzed the regulatory role of PP5 on cdc25/twine in vivo using Drosophila embryos of as a model system.

This is a potentially interesting paper, which is interesting and well written. However, I have one main concern that should be addressed prior publication.

Main point.

1-The analysis of the phosphorylation sites. The authors have identified 8 phosphorylation sites. Only 4 of these seems to actually regulated (directly or not) by PP5. Nevertheless, they have performed mutagenesis analyses on all of these 8 sites. This is difficult to understand and this certainly complexifies the interpretation of the mutant analyses performed since different phosphorylation events may have opposite effects on twine. I therefore strongly recommend to perform analyses of the 4 phosphorylation sites (SA and SD mutants) before publication. I am afraid it is impossible to drive conclusions on the specific regulation of twine by PP5 with the mutants analyzed here.

Minor point.

2-Lane 158: please explain why the half-life of the protein does not account at all for the exponential decay. This is strange as in lane 242, the authors mentioned a decrease of twine half-life. Amend if necessary.

3-What is the viability of embryos with one extra cell cycle ?

4-Is there more twine-GFP protein in most of PP5 mutant embryos in general (Figure 3A suggests it is not the case). I am wondering if the extra-cycle detected here is caused by the fact that there is more twine produced in this context (during oogenesis). This is potentially important as it would suggest that PP5 controls twine levels at earlier steps than cycle 14.

5-Lane 240. Is it documented that twine mRNA is not translated during cycles 1-13 ?
