# Supplementary material for: Fluorescence fluctuation analysis reveals PpV dependent Cdc25 protein dynamics in living embryos
Source: PLoS Genet. 2020 Apr 6;16(4):e1008735. doi: 10.1371/journal.pgen.1008735 (PMC7162543; doi:10.1371/journal.pgen.1008735)
Supplement: Attachment — Submitted filename: Rebuttal Letter.docx [file pgen.1008735.s008.docx]

**Response to Reviewers’ Suggestions**

**(PGENETICS-D-19-01957) - [EMID:71a0ed7420786784]**

**Reviewer #1:** In this paper, the authors have analyzed the regulatory role of PP5 on cdc25/twine in vivo using Drosophila embryos of as a model system.

This is a potentially interesting paper, which is interesting and well written. However, I have one main concern that should be addressed prior publication.

We thank the reviewer’s helpful and supportive comments. We now revise the manuscript accordingly and respond point-by-point below.

Main point.

1-The analysis of the phosphorylation sites. The authors have identified 8 phosphorylation sites. Only 4 of these seems to actually regulated (directly or not) by PP5. Nevertheless, they have performed mutagenesis analyses on all of these 8 sites. This is difficult to understand and this certainly complexifies the interpretation of the mutant analyses performed since different phosphorylation events may have opposite effects on twine. I therefore strongly recommend to perform analyses of the 4 phosphorylation sites (SA and SD mutants) before publication. I am afraid it is impossible to drive conclusions on the specific regulation of twine by PP5 with the mutants analyzed here.

We thank the reviewer for pointing out this. To avoid any misunderstanding and make this finding clear, we have adjusted the text with the description of the phosphosite identification. In fact, we have identified 12 sites in total. 4 of these 12 phosphosites were found in both wild type and *PpV* embryos. The other 8 phosphosites were identified exclusively in *PpV* mutants, indicating that the dephosphorylation of these 8 sites directly or indirectly depends on *PpV* (Fig. 4B). For functional analysis, we generated mutants and corresponding transgenes for these 8 *PpV* dependent phosphosites. We fully agree with the reviewer that different phosphorylation events may have diverse and even opposite effects. We cautiously interpreted and carefully discussed the relation of phosphorylation levels and stability of Twine. We now re-wrote the result part (line 202–210) to better and precisely describe the results of phosphosites mapping. We further discussed this important issue of Twine phosphorylation events and protein activity in the discussion part (line 290–300).

Minor point.

2-Lane 158: please explain why the half-life of the protein does not account at all for the exponential decay. This is strange as in lane 242, the authors mentioned a decrease of twine half-life. Amend if necessary.

Sorry for this confusion. We now added more sentences to clearly distinguish the terms “half life” and phenological “decay time” (line 164–169). ‘Half life’ is generally defined as the molecular lifetime. Our FFA measurements revealed however the steady state levels of the bulk of Twine molecules, whose changes are set by the difference between synthesis and degradation.

3-What is the viability of embryos with one extra cell cycle?

The detailed phenotypic analysis of *PpV* embryos has been presented in a previous research article (Liu et al. 2019, G3). We now slightly expanded the introduction with more information about the mutant phenotype (line 79–83). *PpV* mutant embryos (from *PpV* germline clones) are uniformly embryonic lethal. We did not observe obvious groups of cuticle phenotypes, for example, indicating a link to the cell cycle behavior in blastoderm stage.

4-Is there more twine-GFP protein in most of PP5 mutant embryos in general (Figure 3A suggests it is not the case). I am wondering if the extra-cycle detected here is caused by the fact that there is more twine produced in this context (during oogenesis). This is potentially important as it would suggest that PP5 controls twine levels at earlier steps than cycle 14.

The comment of the reviewer is fully justified, since the *PpV* mutants are embryos from mutant germline clones. Thus, embryonic phenotypes can be based on a function already during oogenesis. To test this hypothesis, we conducted FFA measurements throughout blastoderm cycles as soon as cortical nuclei appeared. We measured nuclear Twine concentrations. Our FFA measurements indicate that in early embryos, Twine levels are similar in wild type and *PpV* mutants, at least in nuclear cycle 11 (Fig. 3A). It is only in cycle 14 that the FFA data are significantly different between wild type and *PpV* embryos.

5-Lane 240. Is it documented that twine mRNA is not translated during cycles 1-13?

We thank the reviewer for raising this important issue. Previous studies (Farrell and O’Farrell 2013, Curr Biol; Edgar and Datar 1996, Genes & Dev) showed that injection of the translation inhibitor cycloheximide into embryos at nuclear cycle 12 resulted no change and into embryos at nuclear cycle 13 resulted in little change of Twine protein levels. Moreover, as the same studies reported, injection of *twine* mRNA into pre-MBT embryos showed increased Twine protein levels at cycle 14, and resulted in a correspondingly shortened cycle 14. These data indicate that *twine* mRNA is translated at least in cycle 13. The current view is that Twine protein is translated throughout nuclear cycles. Evidence of this view is the studies by Di Talia et al. 2013, who employed photo-switchable Twine variants to dissect synthesis and degradation by measuring the molecular half life. These data indicate that Twine protein has a half life of about 20 min in blastoderm cycles (NC11–13) before it abruptly drops during cycle 14. Thus, these data indicate that it is mainly the protein stability (and less so translation) which changes in cycle 14.

**Reviewer #2:** In this manuscript, Liu et al address the mechanisms of cell cycle regulation at the maternal-to-zygotic transition. They focus on the regulation of Cdc25Twine levels, which were previously shown to be essential for cell cycle remodeling. This paper extends our understanding of Cdc25Twine regulation by showing that the levels of protein loaded in the embryo by the mothers increase when Protein Phosphatase V is deleted. As a consequence, a fraction of the embryos would not pause the cell cycle after 13 divisions, but would undergo a 14th mitosis prior to arresting, a typical phenotype observed when the regulation of the maternal-to-zygotic transition is perturbed. These are interesting insights. The authors also show that Twine is hyperphosphoryalted when PPV is deleted. While there are many open questions about the mechanistic action of those phosphorylation the fact that the effects of PPV are through Cdc25Twine phosphorylation (either direct or indirect) will be helpful for future studies. Overall, while many open questions remain on how PPV controls Cdc25Twine during early embryogenesis, collectively the insights presented in this paper are interesting and provide an advancement.

We thank the reviewer’s helpful and supportive comments.

I only have one comment. The authors should quantify the amount of cdc25twine maternal mRNA and compare it to wild type level. It is important to know whether the higher protein levels are also linked to higher mRNA levels. I might have missed this important control- in such case the authors need to discuss it more clearly.

The reviewer is correct that both the transcription and post-transcriptional mechanisms may contribute to Twine protein levels, especially since *twine* RNA vanishes later in cycle 14. We have measured *twine* mRNA levels in early embryos by quantitative PCR. These data are now shown in Figure 1B in the revised manuscript. Our data show no difference between wild type and *PpV* mutants. The data are now described in the text in lines 105–107. We conclude that the part of Twine regulation dependent on *PpV* occurs on post-translational level, consistent with the *twine* RNAi experiments (Farrell and O’Farrell 2013, Curr Biol). We also included this issue in the result and discussion parts (line 99–100, 253–254).

**Reviewer #3:** Originality: To my knowledge the application of FFA to developmentally assess Twe levels and degradation kinetics during cycle 14 is novel and represents an excellent case study for this technology. Previous work indicated that additional factors besides Trbl contribute to Twe destabilization. Evidence that Ppv represents such an additional factor is examined in this study.

We thank the reviewer’s helpful and supportive comments. We now revise the manuscript accordingly and respond point-by-point below.

Importance to field: Unclear. How Cdc25 (both Twe and Stg) phosphatases affect Cdk1 regulation of mitosis during syncytial cycles and the maternal-zygotic transition has been intensively studied in Drosophila.

Our focus is on post-translational mechanisms for the control of Cdc25, which have not been resolved. The papers by Di Talia et al. and Farrell & O’Farrell from 2013 showed that Twine is largely controlled by post-translational mechanisms but left open the mechanisms. Farrell and O’Farrell focused on the function of Trbl as a factor involved in Twine protein degradation, but not exclusive. Given its mutant phenotype is clear, however, that Trbl serves a supporting function besides other yet unknown mechanisms.

The authors propose that Ppv-mediated Twe turnover during late syncytial cycles sets a threshold level that contributes to rapid degradation of Twe during early cycle 14, independently of Trb (or other) mechanisms. Data showing differences in Twe concentration between control and Ppv genotypes and identification of Twe phospho-peptides unique to Ppv mutants is consistent with their model. I don’t see how the experiments with transgenic mutant forms of Twe support this as an essential mechanism, however. Changes in phosphorylation status apparently did not compromise either mutant transgene’s ability to complement twe mutant female sterility and both mutants weakly promoted extra syncytial cycles, as a measure of Twe activity, but it wasn’t that much of an effect.

We appreciate the reviewer’s understanding of the study. Our central arguments in support of our conclusions are the analysis of Twine protein levels and the identification of *PpV* dependent phosphosites. Albeit the transgenic analysis of the phosphosites is consistent with a function of them in control of Twine protein levels, it is clear, as the reviewer states, that it leaves open how these Twine phosphosites function in embryonic cell cycle phenotypes. However, transgenic analysis is an obvious type of analysis that necessarily follows the identification of phosphosites.

We now re-wrote the discussion parts to better interpret the function of Twine phosphorylation status (line 290–300). We stated that the Twine phosphosite mutants are different than the Twine in *PpV* mutants. Both transgenes changed the dynamics of Twine protein. *Twine* homozygous flies with either one copy of the phosphomimic and non-phosphorylatable Twine transgenes are viable and fertile, although the stocks require special attention. Importantly, two copies of the Twine-GFP-8xAsp transgene were lethal. This clearly shows that the induced changes in “phosphorylation status” affected the activity of Twine protein above the physiological level.

Broad interest to genetics and genomics: Potentially this method for dynamically quantitating protein levels in living embryos will be useful for many researchers. The issues they are dealing with are broadly applicable and relevant to understanding other inherently variable processes.

We thank the reviewer’s supportive comments.

Rigorous methodology: Not being familiar with the FFA technique, I cannot comment on that aspect. It wasn’t clear to me how the rate curves were generated, but I appreciated seeing the spread of the individual embryo data. By using a Twe-GFP transgene for these studies in a context of two normal twe alleles the overall Cdc25 levels were effectively increased. Is there a way to account for effects related to Cdc25 activity levels?

We thank the reviewer’s supportive comments. We now better described in the materials and methods part to explain the calculation of the curves and derived parameters (line 361–368). The reviewer raises an important issue, in that the Twine protein levels do not necessarily reflect Twine kinase activity. This issue has not been addressed yet with respect to Twine. Activity could be measured by a kinase sensitive FRET sensor as it is available for Cdk1 (please see studies from Di Talia’s laboratory, for example). Generation of such a sensor would require a significant effort in selection of the target peptide and validation of the construct. We now discussed this issue in the discussion part (line 303–305).

Substantial evidence for conclusions: While the data were convincing, in my view the results were too broadly interpreted to uphold the conclusions stated in the Abstract.

According to the reviewer’s suggestion, we now modified the conclusion part of the abstract and author summary, and carefully checked the results part to ensure our statements are well interpreted.

Minor points:

It seems misleading to say (line 58) that nuclear cycles proceed with ‘ultrafast S phase of about 5 minutes’ when they range from ~2 to 20 minutes long.

We now re-wrote this sentence by saying ‘proceed with fast S phases’.

Needs editorial improvement in places, for example: 36_the pause is occurred.

We now re-wrote this part and checked thoroughly the whole text.

I don’t think you can accurately say (69-70) that ‘only Twe is required for nuclear cycles’, given the conclusions of references [18, 19].

We now re-wrote this sentence by saying ‘Twine is specifically required for the nuclear cycles’.

Wee1 phosphorylates Cdk1 on Y15. The Wee1-related Myt1 kinase phosphorylates Cdk1 on T14 and Y15.

We now corrected this content to Wee1/Myt1.

Fig 1B/C: the evidence that Twe disappears more slowly in Ppv mutants vs WT in cycle 14 by IF looks convincing (for the two embryos shown) but the westerns showed little difference between these genotypes. Should the western data be discounted because of inherent difficulty with normalizing embryo lysates? High embryonic variability seems to be a feature of Ppv mutant embryos, making it difficult to draw conclusions from these comparisons.

The reviewer is fully right. We would like to keep the western blot and the embryo IF staining in the manuscript to illustrate the need for a non-invasive method with high and clearly defined temporal resolution. We added more explanation in the result part (line 113–114).

Fig 4. The proteomics analysis of Twe-GFP shows a big difference by NC11 in Twe concentration between the different transgenes. Phospho-mimetic mutant has much lower levels at all time points – does this mean the changes are destabilizing the protein but also making it refractory to further loss below this threshold? Phospho-acceptor mutant starts higher, but seems to decline at a similar (?) rate as WT. Yet both transgenes rescued twe mutant female sterility. Do these results really support Ppv-mediated turnover of Twe being important for MZT kinetics?

As mentioned above, we now modified our discussion according to this issue (line 290–300, line 303–305). As stated now in the discussion part, we do not know how the changes of phosphorylated residues alter Twine activity. The mutated residues may affect kinase activity, substrate specificity or interactions with other proteins. It is also conceivable that Twine has to undergo a reaction cycle of successive and transient phosphorylation and dephosphorylation. We calculated a decay rate only for interphase 14 but not for the short earlier cycles. The transgenic analysis is not needed for the central conclusion that Twine protein levels depend on *PpV*. The transgenic analysis is however a necessary step following the phosphosite identification.
